# Transient Effects of Applying and Removing Strain on the Mechanical Behavior of Rubber

**DOI:** 10.3390/ma13194333

**Published:** 2020-09-29

**Authors:** Elli Gkouti, Burak Yenigun, Aleksander Czekanski

**Affiliations:** Department of Mechanical Engineering, Lassonde School of Engineering, York University, Toronto, ON M3J 1P3, Canada; byenigun@yorku.ca (B.Y.); alex.czekanski@lassonde.yorku.ca (A.C.)

**Keywords:** relaxation, recovery, viscoelastic materials, rubber, finite element model

## Abstract

For viscoelastic materials, the relationship between stress and strain depends on time, where the applied strain (or stress) can be expressed as a step function of time. In the present work, we investigated two temporary effects in the response of viscoelastic materials when a given strain is applied and then removed. The application of strain causes a stress response over time, also known as relaxation. By contrast, recovery is the response that occurs following the removal of an applied stress or strain. Both stress and relaxation constitute transient stages of a viscoelastic material exposed to a permanent force. In the current work, we performed several experimental tests to record the recovery in response to the total or partial removal of the strain. By observing and analyzing the mechanical response of the material to strain, we deduced that recovery is a procedure not only related to creep but also to relaxation. Hence, we created a model that simulates the behavior of viscoelastic materials, contributing to the prediction of relevant results concerning different conditions.

## 1. Introduction

In materials science, several phenomena have been characterized by defining the relationship between a force that causes deformation from the initial conditions and the material’s response to that force. The deformation is due to a stress or strain application that affects the stress or strain conditions, respectively. The simplest cases of deformation involve ideal elastic materials [1] and viscous fluids, whereby the relationship between stress and strain is described by Hooke’s law of elasticity and Newton’s law of viscosity [2], respectively. However, in reality, most materials include physical phenomena that exhibit both elastic and viscous behaviors, and thus a conjunction theory is required. Viscoelastic theory provides a more accurate approximation of the material behavior in this case, as it describes the relationship between stress and strain with respect to time [3].

Owing to their mechanical properties, viscoelastic materials can exhibit complex behaviors in diverse engineering contexts. For many decades, materials with viscoelastic properties have been used in manufacturing and construction [4], bioengineering [5,6], and even toys [7]. The most recent concern among engineers has been understanding the mechanical behavior of these materials in order to predict their response to deformation under various conditions. Hence, performing experimental tests in rubbers under several conditions, contributed excessively to the understanding of their mechanical response. As a result, theoretical methods that were developed are compared with experimental tests including rubbers. It was observed that rubbers subjected to large deformation exhibited a nonlinear behavior, and, consequently, more complicated methods were required for modeling their response. A theoretical framework which includes nonlinear constitutive parameters, such as shear modulus and Poisson function, was developed and defined by comparisons between axial and shear experimental tests [8,9,10]. The relaxation modulus is also required for modeling the viscoelastic behavior of materials. In a recent study, for defining the relaxation modulus of high viscosity asphalt sand, a relative method was developed based on the three-point bending creep test. According to this method, the relaxation characteristics of the asphalt mixtures can be studied effectively [11]. Moreover, rubbers exhibit dynamic characteristics, which are significant for applications in automobiles, machines, and buildings. Static and dynamic tests showed that not only temperature and frequency but also pre-strain has a huge impact to the viscoelastic properties of rubbers [12]. By performing dynamic compression experiments, it was observed that the storage modulus increased with increasing pre-strain and the loss modulus is decreased by decreasing pre-strain. A high priority is also the interpretation of some materials’ tendency to recover after deformation (e.g., in response to temperature change and healing after crack propagation or fatigue) [13,14,15]. More specifically, at room temperature and in response to low strain, the mechanical behavior of common metals such as steel or aluminum as well as that of quartz does not deviate much from the classic theory of linear elasticity and therefore can be described by Hooke’s law. However, for large deformations or at high temperature, materials such as synthetic polymers, wood, metals, and human tissues exhibit significant viscoelastic behavior and thus require a more complicated analytical treatment [16]. In some applications, even a small viscoelastic response can have a significant impact on the material’s behavior. Hence, an analysis or design involving such complex materials must incorporate their viscoelastic behavior to be sufficiently accurate.

**Figure 1:**
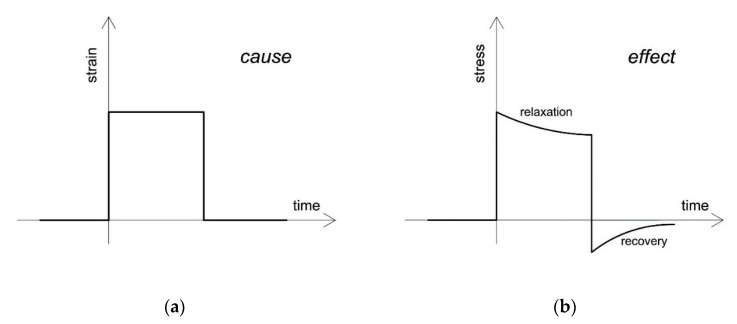
Relaxation and recovery. (**a**) The history of applying and removing strain over time (cause). (**b**) The stress response following the application and removal of strain as a step function of time (effect).

Awareness of the viscoelastic response of a material offers important information concerning the material’s mechanical behavior. To observe this response, several experimental tests must be done. For our purposes, several experiments were performed on natural rubber, a representative viscoelastic material. The mathematical formulation of viscoelasticity theory is presented in the following section with the aim of enabling the prediction of the material’s response to arbitrary strain histories. When testing and describing these materials, it is preferable to apply a step strain or step stress in time rather than a ramp (constant rate of strain or stress) because the effect of time is then isolated from any nonlinearity. The response to step strain is stress relaxation, and the response to step stress is creep (Figure 1).

The viscoelastic theory was studied thoroughly in the past decades and constitutive models were developed [17,18]. Considering the theory, several investigations have been carried out on creep and its recovery [19,20,21]. Some researchers have experimentally studied the response of relaxation, creep, recovery, and hysteresis and related the rates of these procedures to each other [19,20]. Furthermore, experimental research has been done including temperature effects, especially in creep recovery [21]. Alternatively, the stress response to reducing the strain applied to natural and high-damping rubber parts has been investigated [22]. Viscoelastic relaxation can occur whenever there is a delayed rearrangement of the internal structure of the material under stress. There are many ways in which this rearrangement can occur. By understanding viscoelastic mechanisms, situations can be anticipated where viscoelasticity is expected [3].

Thus, analyzing recovery is important not only for simulating and predicting the unloading procedure of viscoelastic materials, but also for understanding their mechanical response to subsequent loading. Repeated loading–unloading procedures are extremely common in real-world applications and therefore simulating them is mandatory. Essentially, if recovery is not completed, residual strain (or stress in the case of creep) will without question affect subsequent loading, as the material does not begin from the anticipated conditions. Consequently, several errors will affect prediction of the mechanical response to future loading–unloading, and hence an incorrect strain history will cause an incorrect response to simulation models. As previously mentioned, several studies have been done to understand the stress relaxation and creep behavior of different materials. However, few studies have investigated the recovery periods following stress relaxation, resulting in a limited understanding of the viscoelastic behavior of rubber.

Rubber is a representative viscoelastic material owing to its unique mechanical behavior, which differs from that of any other ordinary hard solid. The behavior of rubber can be determined based on its composition, and its constituent molecules can be analyzed according to several theories. Currently, it is possible to simulate even the influence of the thermal agitation of the atoms in the chains, predict nonlinearities, and understand their inner interaction with embedded materials.

In the present work, we have devoted our attention to analyzing the phenomenon of stress relaxation and its recovery in natural rubber. We performed experimental tests in order to record and analyze the mechanical behavior of natural rubber during several loading cycles with relaxation and recovery. After analyzing the results, we created a Finite Element Model (FEM) in a commercial software (ABAQUS) in order to simulate the transient effects of applying and removing strain. Finite element is a common solver of problems related to the mechanical behavior of materials and, recently, it has been widely used [23,24,25]. The adequate results of simulating these responses show that both relaxation and recovery can be modeled with accuracy and hence are possible to be described by the viscoelastic theory, as commercial software, such as ABAQUS, uses time domain constitutive equations of viscoelasticity [26].

## 2. Materials and Methods

**Figure 2:**
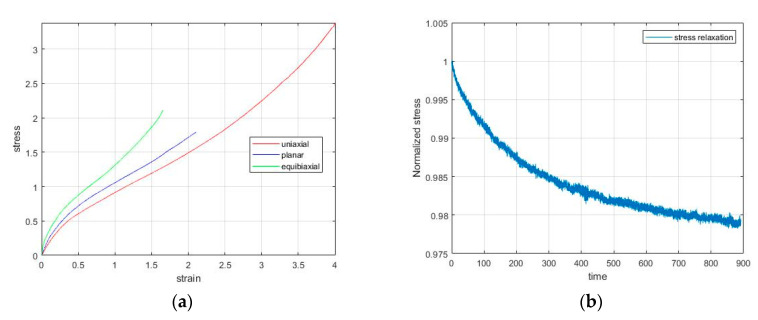
Experimental data of (**a**) uniaxial, planar, equibiaxial tests until fracture, (**b**) stress relaxation beginning from 100% strain.

In order to analyze and simulate the viscoelastic behavior of natural rubber, we first performed several experimental tests on different coupons with a so-called dog-bone shape (115 mm × 25 mm × 3.175 mm). Specifically, rubbers were subjected to uniaxial, planar, equibiaxial tension until fracture, as shown in Figure 2a. Natural rubber is considered to be nearly incompressible and so the volumetric response can be defined by the Poisson ratio, which is almost 0.5. For observing the viscoelastic behavior of the rubber, a stress relaxation test was performed. Namely, the material was subjected to uniaxial tension until it reached 100% of its strain and then the strain was held constant for 900 s. We observed that the respond to that cause was the stress relaxation for as long as the strain was held constant, meaning that the stress started to relax until it asymptotically reached a specific stress value. Figure 2b shows the experimental results concerning the stage of stress relaxation for 100% strain.

To verify the accuracy of the viscoelastic analysis of the rubber, a specific small area (5 mm) in the center of the coupon was marked by two pieces of tape. For performing the experimental tests that were mentioned above, one of the sample’s edges was held constant and the other was free to extent. Then, during the extension of the coupon, the displacement for both this area and the crosshead were measured by an extensometer of 100 mm capacity and the MTS machine, respectively [27]. Alternatively, to measure both axial and lateral dimensions of a delimited area of a sample, a DIC machine can be used [28,29]. The experiments were conducted in a quasi-static strain rate with the crosshead moving 0.1 mm/s, with a sampling rate of 5 Hz at constant room temperature. The results were then analyzed and material parameters were evaluated in order to be used as the input to create FEMs in a commercial software (ABAQUS) (ABAQUS 2020X, Dassault Systèmes, France).

**Figure 3:**
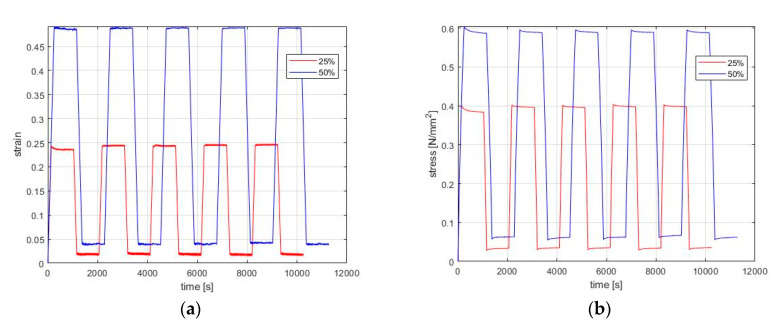

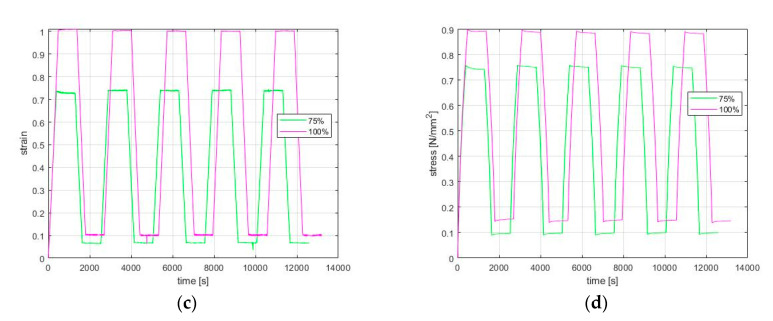
Five repeating cycles of loading–unloading with relaxation and its recovery for different strain levels. Graphs in the left column show the history of applying (**a**) 25%, 50%, (**c**) 75%, and 100% and partially removing the strain until it reaches 10% of the applied strain over time (cause). Graphs on the right column show the stress response following the application and removal of (**b**) 25%, 50%, (**d**) 75%, and 100% as a step function of time (effect).

The purpose of the present research is to show that rubbers exhibit a transient behavior similar to relaxation, when a deformation is removed, known as recovery. We performed several experimental tests of 5 repeating cycles of loading–relaxation–unloading–recovery for different strain levels in order to record this behavior (Figure 3). Until now, many papers included experimental results and simulations of loading, relaxation, and unloading stage in a commercial software [30,31,32]. ABAQUS is usually used for simulating viscoelastic behavior with accuracy, as it deals with nonlinearities that are entering when large deformations are applied. As in our case, the deformation is large (greater than 20% of strain) and so the relationship between stress and strain is nonlinear.

Hence, the simple equation of Hooke’s law is not applicable and thus a more complicated theory is required. Hyperelasticity uses the strain energy density function in order to calculate the stress tensor, which has the following form

(1) $$\sigma_{j}=-p+\lambda_{j}\frac{\partial W}{\partial_{\lambda j}},$$

where $\lambda_{j}$ are the principal stretches, *p* is the pressure, and *W* is the strain energy density function. For defining the strain energy density function, several models have been developed such as the neo-Hookean, Mooney-Rivlin, polynomial, and Ogden model [33]. We compared all the models mentioned above with the experimental results, shown in Figure 2a, and the only stable model that fitted better was the Ogden model with one term (*N* = 1), which is given by

(2) $$W(\lambda_{1},\lambda_{2},\lambda_{3})=\frac{2\mu_{1}}{a_{1}^{2}}(\lambda_{1}^{a_{1}}+\lambda_{2}^{a_{1}}+\lambda_{3}^{a_{1}}-3),$$

where $\lambda_{1},\lambda_{2},\lambda_{3}$ are the principal stretches and $\mu_{1},\;a_{1}$ are material constants. In our work, we used the test data of stress-strain, shown in Figure 2a, as an input to ABAQUS in order to calculate the material parameters $(\mu_{1}=0.554428850,$$a_{1}=2.13785974).$ Since the number of stress equations is greater than the number of unknown constants, a least-squares fit was performed to determine the hyperelastic constants. It is obvious that instead of ABAQUS, another software can evaluate the material parameters with accuracy. Moreover, the hyperelastic model can be used for describing not only the loading but the unloading stage as well [34].

Since the hyperelastic model was created and the instantaneous shear modulus was calculated, the viscous effects can be modeled. When strain is applied to the material, the response is the stress. Since natural rubber is a nearly incompressible material, only shear (deviatoric) behavior occurs. Hence, the following constitutive equation shows the relation between shear stress and the time varying shear strain [17,26]

(3) $$\tau (t)=\int_{0}^{t}G_{R}(t-s)\dot{\varepsilon }(s)ds,$$

where $G_{R}$ is the time-dependent “shear relaxation modulus” that characterizes the material’s response and ε(s) is the time-varying shear strain. The upper dot represents the time derivative of the strain. ABAQUS assumes that the viscoelastic material is defined by a Prony series expansion of the dimensionless relaxation modulus provided by

(4) $$G_{R}(t)=G_{0}\left[1-\sum_{i=1}^{N}g_{i}(1-e^{-t/\tau_{i}})\right],$$

where $g_{i},\tau_{i}$ are material constants and i=1,2,…,N is the number of terms in the Prony series. Although there is no limitation on the number of terms that can be used, we selected 1 term (*N* = 1) since the model provides adequate results. We defined these parameters by including the relaxation test data, shown in Figure 2b, directly to ABAQUS and setting 0.002 as the allowable average root-mean-square error. For further theoretical analysis, online documentation of ABAQUS is available [26]. In order to avoid directly using the test data in the future, the evaluated Prony series parameters can be used $(g_{1}=0.007662,$$\tau_{1}=212,11).$ Consequently, the mechanical behavior of natural rubber is modeled and hence it can be used for simulating similar deformations under several conditions by applying the desired boundary conditions and a proper mesh size.

However, the purpose of the current work was to study the reaction of natural rubber to the transient effects of applying and removing a strain. For this purpose, a set of experimental tests was performed under repeating conditions. A dog-bone coupon of natural rubber was subjected to uniaxial loading until it reached zero strain at which point it was held constant for 900 s (relaxation). After that time, the load was released until it reached zero strain, and it was then held constant for another 900 s (recovery). The above experiment was repeated four times after the end of the first recovery procedure. All experiments were performed under the same conditions as before (0.01 mm/s speed, room temperature, sampling rate 5 Hz).

The experimental procedure was then repeated, but the applied strain was not totally removed as before in order to avoid the folding of the coupons. Instead, during this experiment, the focus was on investigating relaxation and its recovery. Therefore, strain was kept constant at 10% of the previous strain level for 900 s. Figure 3 shows the experiments for 25%, 50%, 75%, and 100% applied strain, which was kept constant, and where the relaxation responded; the recovery started after partly removing the applied strain. As before, both experiments were repeated four times after the end of the first recovery at room temperature, and the strain rate was as before.

A phenomenon associated with stress softening, the Mullins effect, must be accounted for to meet the requirements of the present research [34,35,36,37]. Namely, when a rubber sample is extended from its virgin position, unloaded, and then reloaded again, the required stress upon reloading is less than that needed at the first loading for strains up to the maximum strain reached on the initial loading. After some cycles, the stress level is observed to reach an almost constant value [36].

Due to the limitation of similar results concerning the stage of recovery, when we observed the results (Figure 3), we speculated that this stage is the “opposite” procedure of stress relaxation as the rubber continues to respond as a viscoelastic material. Based on the theory [3], when a strain is applied until it reaches a desired level (cause) and then it is held constant for a specific time period, then the stress relaxes for this period (effect), until it asymptotically reaches a stress value. Namely, relaxation is a transient effect that lasts as long as the strain is held constant. After loading and relaxation, the material is free to unload, and returns to its initial position. However, unloading is also a cause of the material’s respond, as the strain is removed and then it is held constant for almost zero strain for the same time period as the relaxation. Namely, unloading causes the recovery, and as a result, the viscoelastic behavior of the material continues after unloading as the rubber tends to return to its initial state. This behavior was observed in the experimental results, but the question was if the same equations of viscoelasticity [17] can be used. Since a commercial software, such as ABAQUS, uses the theory that describes the viscoelastic behavior of the rubber [17,26], we used the viscoelastic model that we have created in order to simulate recovery under the same conditions that the experiments were performed. As it was mentioned above, relaxation recovery is the effect of removing a strain, so strain must be applied first. As a result, we had to simulate the entire cycle loading–relaxation–unloading–recovery. For simulating every cycle of loading–relaxation–unloading–recovery, we designed a 3D solid part with the same dimensions of the dog-bone sample that we have used for the experiments. For more accurate results, we used 0.5 as a mesh size. The element type was C3D8 which is appropriate for nearly incompressible materials that are subjected to large deformation. The boundary conditions were the same as the experiments, namely, one edge was held constant and the other was free to extend. For simulating loading and unloading, a “static” step was used, while for relaxation and recovery, a “visco” step was applied. The time period for the “static” steps was in accordance to the constant strain rate (0.02 s^−1^) and for the “visco” step it was 900 s, as in the experiments.

## 3. Results

### 3.1. Experimental Test Results

The transient effects in natural rubber were obvious for both series of experiments. The curve of relaxation is relative to the strain level reached during loading. The same holds true for the case of recovery. In Figure 3, it is obvious that when relaxation ends, stress asymptotically reaches a specific value. The same was observed for recovery as well. That means that the material is partially “resting” and, as a result, the effect of strain is not vanishing. The material needs time to be “healed” properly and thus it keeps “working” for recovery. If the material is not returning to its initial position, then the residual displacement constitutes a plastic behavior of the material [34]. In order for the “healing” procedure to be more rapid and complete, other techniques should be followed, primarily using heat treatment [21].

Moreover, for all cases of repeated loading cycles shown in Figure 3, the stress softening was obvious for all strain levels. In the initial loading, the required stress is always more than the amount needed for the subsequence cycles. As was anticipated [34], for a few cycles after the virgin loading (in this case, three or four), the stress levels required for the relaxation to begin almost coincided for all cases of strain. Different values of stress were also observed when the recovery began. As Figure 3b,d shows, the minimum stress required for the recovery to begin differs between the initial cycle and the following cycles. As was the case for relaxation, after some cycles, the stress level is almost the same when recovery starts. As was expected, for both transient effects, the level of stress softening was greater as the strain increased.

### 3.2. FEM Compared with Experimental Results

As was mentioned above, the FEM was designed by using the experimental results of the tests on the hyperelastic and viscoelastic behavior of natural rubber as input. Specifically, to simulate the viscoelastic behavior of the rubber under the same conditions as we did for the experiments, a strain history was designed for the model. For each strain case, four different steps were used to describe loading, relaxation, unloading, and recovery, following the same time and strain rate as was used in the experiments. Stress and strain results were also exported for the small area in the center of the model (5 mm) for comparison with the relevant experimental results.

**Figure 4:**
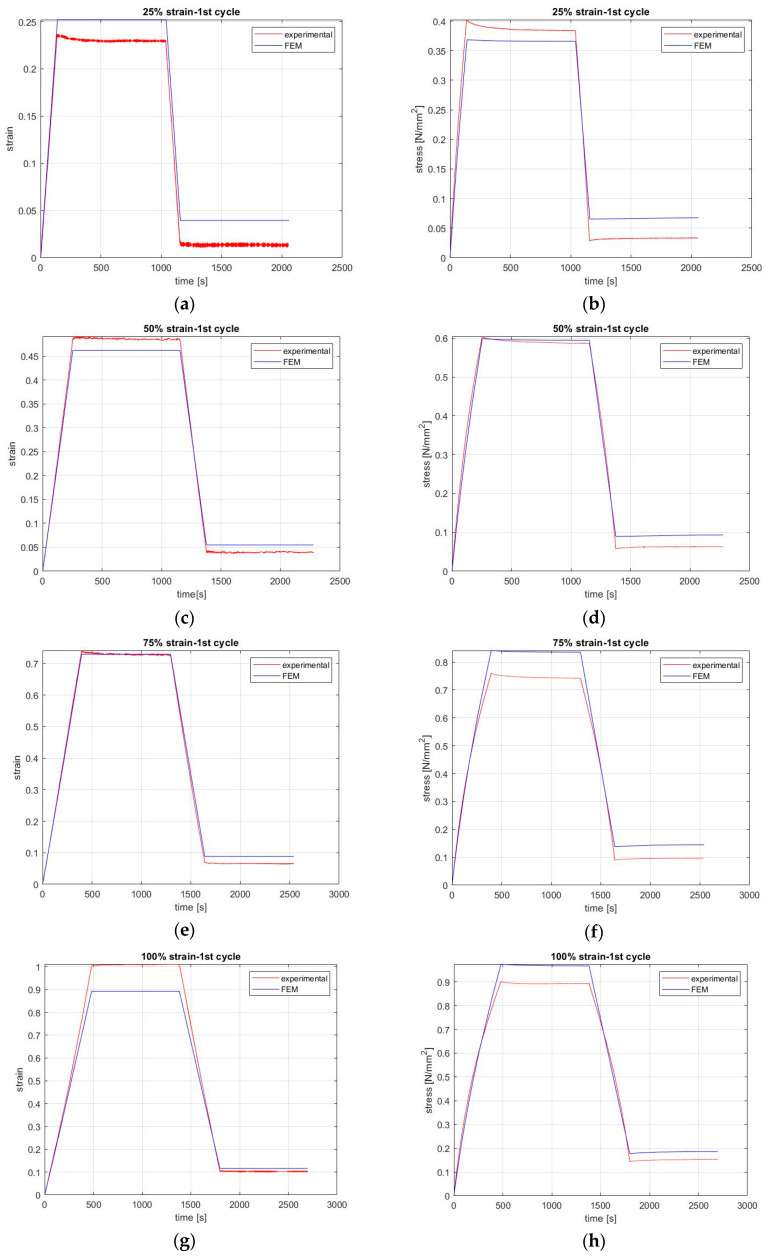
FEM compared with experimental results for the first cycle. Graphs in the left column show the history of applying (**a**) 25%, (**c**) 50%, (**e**) 75%, and (**g**) 100% strain and partially removing it until it reaches 10% of the applied strain (cause). Graphs in the right column show the stress response following the application and removal of (**b**) 25%, (**d**) 50%, (**f**) 75%, and (**h**) 100% strain as a step function (effect).

**Figure 5:**
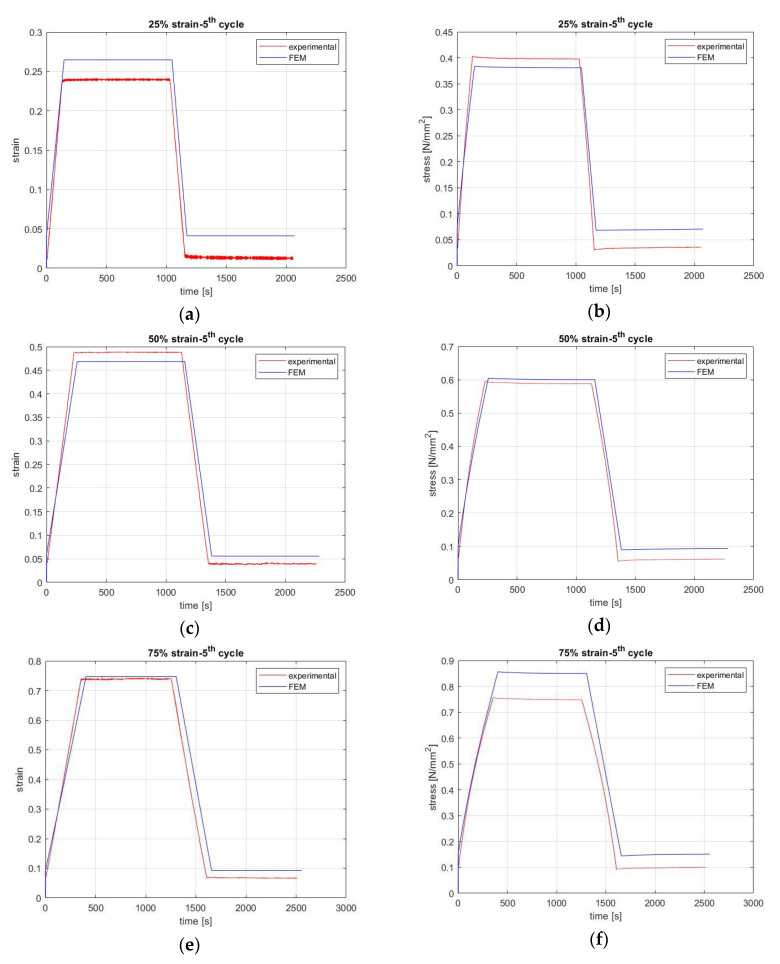

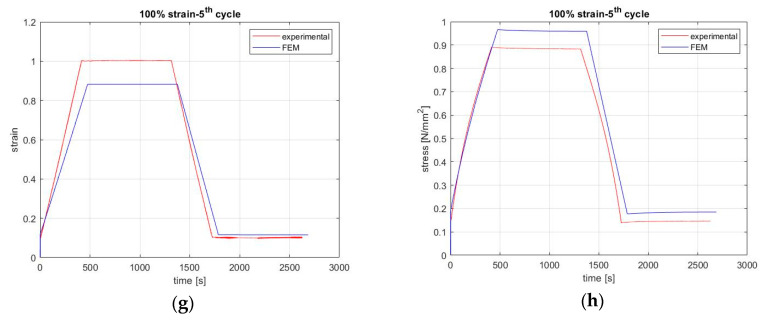
FEM compared with experimental results for the fifth cycle. Graphs in the left column show the history of applying (**a**) 25%, (**c**) 50%, (**e**) 75%, and (**g**) 100% strain and partially removing it until it reaches 10% of the applied strain (cause). Graphs in the right column show the stress response following the application and removal of (**b**) 25%, (**d**) 50%, (**f**) 75%, and (**h**) 100% strain as a step function (effect).

The comparison between the created model and the experiments is shown in Figure 4 and Figure 5 for all strain levels during the first and fifth cycle, respectively, as they are the most representative cases for showing the effect of stress softening in rubber. Specifically, the first cycle shows the virgin state of the coupon and the fifth shows the stress value after stress softening has occurred. The history of applying and partially removing different levels of strain is shown in the left column, and the stress response to these causes is shown in the right column of Figure 4 and Figure 5. The FEM results are sufficiently close to the experimental results, as the error is less than 11%. For the case of 75% strain, the error is 11%, but in the case of 50% strain, the error is almost 1% for all cycles.

**Figure 6:**
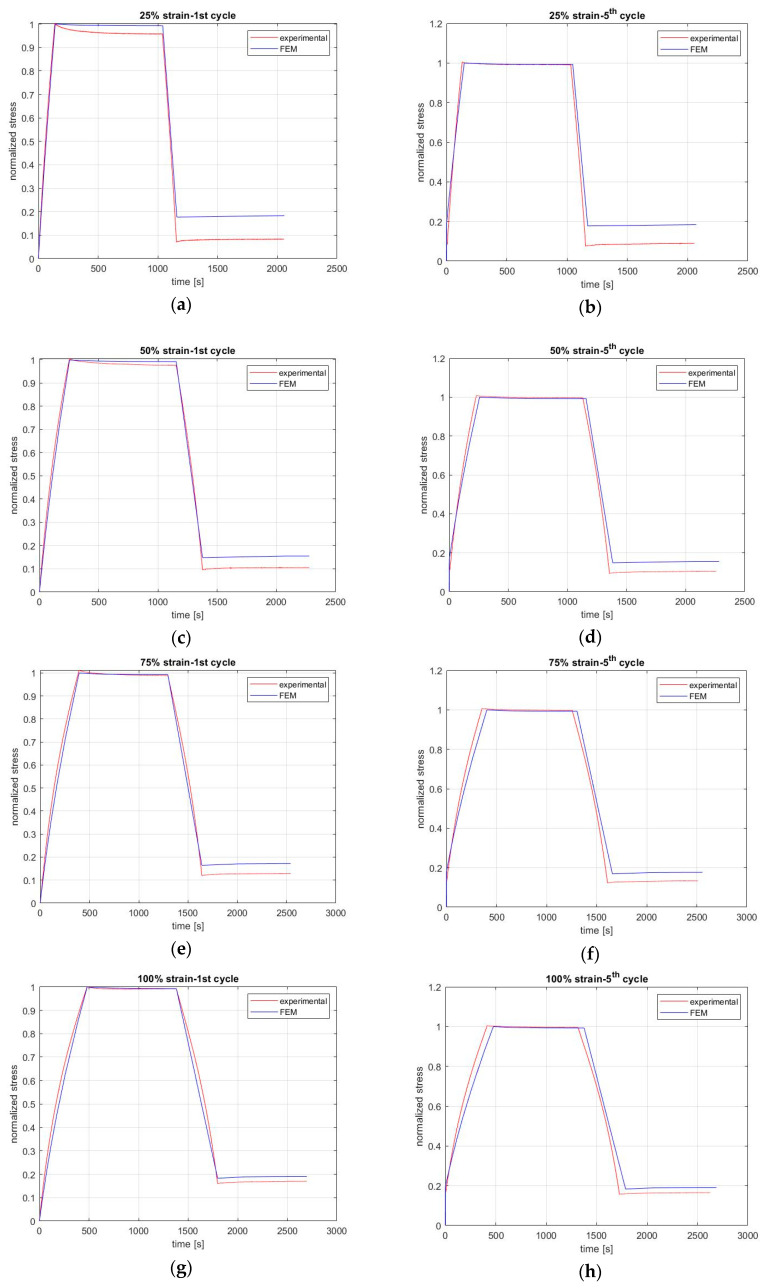
The normalized stress response following the application and removal of strain as a step function of time during the first cycle (left column) for the strain levels of (**a**) 25%, (**c**) 50%, (**e**) 75%, (**g**) 100% and fifth cycle (right column) for the strain levels of (**b**) 25%, (**d**) 50%, (**f**) 75%, (**h**) 100%.

Figure 6 illustrates the change in normalized stress over time, where the accuracy of the designed FEM is adequate. Every absolute value of stress is shown in Figure 4; Figure 5 is divided by the maximum stresses that the rubber captured when relaxation began. In the left column of graphs in Figure 6, the stress response to the applied and partially removed strain values is shown for the first cycle, and in the right column of graphs, the relevant results are presented for the fifth cycle.

## 4. Discussion

Our experiment results show that both the relaxation and recovery of natural rubber can be well approximated by a power law as a function of time. Namely, recovery is a viscoelastic response to the cause of removing a deformation and can be adequately modeled by a commercial software. The model that we have created in ABAQUS is capable of simulating the behavior of rubber for a complete cycle of loading with relaxation and its recovery, although some differences still remain. During the experiments, the displacement is recorded for the whole sample by using the crosshead of the MTS machine and also for the small area (5 mm) in the center of the sample by a laser extensometer. For the model, the data of the crosshead are used for creating the step history of the material’s deformation and the response is predicted for both areas. With respect to strain levels, the differences are mainly due to the incorporating nonlinearities of the material.

However, differences could also be attributed to the slippage of the coupon in the grips, the composition of the rubber, and, of course, to the inadequacy of the FEM, which is related to the nonlinear behavior of natural rubber. More specifically, the test coupons were cut from material that is known to lack uniformity inside, and hence their composition differs, especially if the rubber is filled. Namely, the position of the embedded material is arbitrary, and so the chains between them and the material vary in length and strength [38]. The error between the experimental and FEM results was less than 11% for both the first and fifth cycle. As is obvious from Figure 4 and Figure 5, the case of 75% strain has the worst accuracy (11%). In other words, the absolute values are not exactly the same, and hence this is an area that must be improved.

On the other hand, if we are interested only in the stress change with time according to the same rate for both the experimental and FEM results, the normalized stress versus time can be used. As shown in Figure 6, the results show better accuracy for all strain levels for the first and fifth cycles. Especially for loading and relaxation, the similarity between the FEM results and experimental results is adequate. However, for unloading and recovery, there are differences that are due to the delay in recovery of the rubber. As previously mentioned, to build the FEM, the same strain rate was used for both loading and unloading. However, in reality, the material needs more time to begin recovery, and thus the unloading lasted longer than predicted based on the constant strain rate. As a result, the minimum stress needed for the recovery to begin was less in the experimental than in the FEM. Despite this delay, it is obvious that stress changes in the same way for all cases of recovery, as shown in Figure 6.

## 5. Conclusions

Stress relaxation and recovery constitute the permanent effects of applying and removing a strain to a material, respectively. These two stages are essentially opposite, as relaxation is the result of applying a strain, and recovery is the consequence of removing it. Viscoelastic materials can be investigated while exhibiting these effects, and the results of such a study are useful for simulating, designing, and ultimately predicting the mechanical behavior of materials. If materials such as rubbers can be modeled to simulate deformation under the desired conditions, then the same is possible for the recovery procedure of the material’s returning to its initial position.

Moreover, it is mandatory to be able to predict several cases, including the transient effects on rubber, which are common in many fields, as it is costly and time consuming to perform experiments every time conditions change. We can speculate that for cases where the stress is kept constant after unloading, creep recovery can be performed with great accuracy, as it is a procedure relevant to the case discussed in this research [20]. More specifically, after unloading—where zero stress is reached—creep recovery begins and continues for as long as the stress is held constant. The FEM that we have created can be used for simulating and predicting future loading cycles with relaxation and recovery under several conditions, including creep recovery.

The FEM that we have created is capable of capturing both transient effects relatively well, as the error is 11% at most. As a result, the theory that describes the viscoelastic behavior of natural rubbers and is used by a commercial software, such as ABAQUS, can simulate the stage of recovery rather well. However, the difference between the absolute values for the experimental results compared with the FEM results arose for several reasons, especially those pertaining to the composition of the rubber. Consequently, one way to limit the error between the experimental and FEM results is to perform several tests under the same conditions using different coupons and save these results to a database. From there, we can take the average value of the strain of a sample measured in the crosshead. By considering the strain rate, the calculation of the time that the material needs to achieve the desirable strain is trivial. In other words, the step function needs to be defined to simulate the material’s response by holding the strain constant afterwards.
